# Effect of Pulmonary Vein Isolation with Left Atrial Wall Isolation Plus Selective CFAE Ablation in Patients with Persistent Atrial Fibrillation

**DOI:** 10.3390/jcdd11100308

**Published:** 2024-10-04

**Authors:** Yang Pang, Tao Yu, Ye Xu, Qingxing Chen, Yunlong Ling, Guijian Liu, Kuan Cheng, Junbo Ge, Wenqing Zhu

**Affiliations:** Department of Cardiology, Shanghai Institute of Cardiovascular Diseases, Zhongshan Hospital, Fudan University, Shanghai 200032, China; 851017py@163.com (Y.P.); 23111210146@m.fudan.edu.cn (T.Y.); xu.ye@zs-hospital.sh.cn (Y.X.); chen.qingxing@zs-hospital.sh.cn (Q.C.); ling.yunlong@zs-hospital.sh.cn (Y.L.); liu.guijian@zs-hospital.sh.cn (G.L.); cheng.kuan@zs-hospital.sh.cn (K.C.)

**Keywords:** atrial fibrillation, PWI, CFAEs, ablation

## Abstract

Background: Pulmonary vein isolation (PVI) is a foundational treatment for persistent atrial fibrillation (PeAF), but the effectiveness of adding posterior wall isolation (PWI) and selective complex fractionated atrial electrogram (CFAE) ablation in the roof and anterior wall remains debated. The potential of these additional ablation techniques to improve long-term outcomes for PeAF patients is still uncertain. Methods: This retrospective study included 151 PeAF patients who underwent first-time catheter ablation at our center. The choice of ablation strategy was based on the operator’s clinical judgment, taking into account the patient’s specific condition and anatomical features. Patients were divided into two groups: the PVI group, which received PVI alone, and the modified PWI (MPWI) group, which received PVI along with additional PWI and selective CFAEs ablation in the roof and anterior wall. The primary endpoint was the absence of atrial arrhythmia lasting more than 30 s, without antiarrhythmic drugs, at 12 months. Results: At the 12-month follow-up, 77.3% of the patients in the MPWI group and 52.1% of the patients in the PVI group remained in sinus rhythm without an atrial arrhythmia recurrence (*p* = 0.001). The BIC-based Cox regression analysis identified the ablation strategy and atrial fibrillation (AF) duration as independent predictors of recurrence across the cohort. It was found that MPWI significantly reduced the risk of recurrence, while a longer AF duration increased it. In the MPWI group, AF duration, left ventricular internal diameter in systole (LVIDs), and moderate or greater tricuspid regurgitation were independent predictors of recurrence. In the PVI group, only the left atrial low voltage area (LVA) index was a significant predictor. Conclusion: The addition of PWI and selective CFAE ablation to PVI significantly improves 12-month arrhythmia-free survival compared to PVI alone, demonstrating the superiority of this combined approach in improving long-term outcomes for patients with persistent AF.

## 1. Introduction

Pulmonary vein isolation (PVI) is the guideline-recommended cornerstone for the ablation of symptomatic paroxysmal and persistent atrial fibrillation (PeAF) [1,2,3]. However, the success rate for PeAF has been significantly lower than that of paroxysmal atrial fibrillation, as reported by previous studies [4]. Various adjunctive strategies have been investigated to improve ablation outcomes, but none have consistently demonstrated superiority over PVI alone in large randomized clinical trials [5,6].

The posterior wall of the left atrium is embryologically related to the pulmonary veins and has been proposed to play a role in the maintenance of persistent AF [7,8,9]. Several nonrandomized studies have shown promising results [8,9,10,11], whereas the CAPLA (Effect of Catheter Ablation Using Pulmonary Vein Isolation with vs. without Posterior Left Atrial Wall Isolation on Atrial Arrhythmia Recurrence in Patients with Persistent Atrial Fibrillation) trial reported no improvement in outcomes compared to PVI alone. However, a posterior low-voltage area (LVA) was associated with a significant increase in atrial arrhythmia recurrence [12,13]. The negative result of the CAPLA study was thought to be due to the presence of more extensive atrial substrate beyond the posterior wall.

Several large randomized studies have also demonstrated no improvement when LVA ablation was added to PVI in patients with atrial fibrillation [5,14,15]. These studies did not show any significant benefit of LVA ablation over PVI alone in reducing arrhythmia recurrence. Furthermore, LVA mapping in most studies was performed during sinus rhythm rather than during atrial fibrillation [5,14,15]. This mapping strategy may underestimate the extent of electrical remodeling in the cardiac tissue and result in excessive ablation in the left atrium (LA). Complex fractionated atrial electrograms (CFAEs) recorded during AF may represent critical areas responsible for maintaining AF [16,17]. Selective CFAE ablation could target AF triggers and modify the substrate responsible for its perpetuation.

In this study, the influence of substrate identified during atrial fibrillation, rather than sinus rhythm, on PeAF ablation outcomes was examined. A modified PWI strategy (PVI plus PWI and selective CFAE ablation in the anterior/roof wall of the LA) was compared to PVI alone through a retrospective analysis.

## 2. Method

### 2.1. Study Population

This investigation was a retrospective, nonrandomized, single-center analysis. A total of 151 consecutive patients with symptomatic persistent atrial fibrillation (PeAF), resistant to at least one antiarrhythmic medication, were included between November 2020 and December 2022. Participants were 18 years of age or older, had symptomatic persistent atrial fibrillation (defined as a sustained episode lasting more than 7 days) that was unresponsive to at least one antiarrhythmic drug, and were undergoing catheter ablation for the first time. Patients were excluded if they had paroxysmal atrial fibrillation, atrial fibrillation lasting more than 3 years, a left atrial diameter of 60 mm or greater, structural heart disease, or other severe medical conditions. Detailed inclusion and exclusion criteria are provided in Table S1 in the Supplementary Material. All participants provided written informed consent prior to enrollment.

Patients stopped taking antiarrhythmic medications for at least five half-lives before undergoing their first ablation at our center. They were allocated to either the MPWI or the PVI-only groups based on clinical judgment and individual preference. This non-randomized allocation was determined by the physicians considering factors such as patient characteristics, anatomical considerations, and clinical experience, reflecting real-world decision-making in PeAF treatment strategy selection. The study protocol received approval from the Ethics Committee of Zhongshan Hospital, Fudan University.

### 2.2. Ablation Procedures

**Figure 1:**
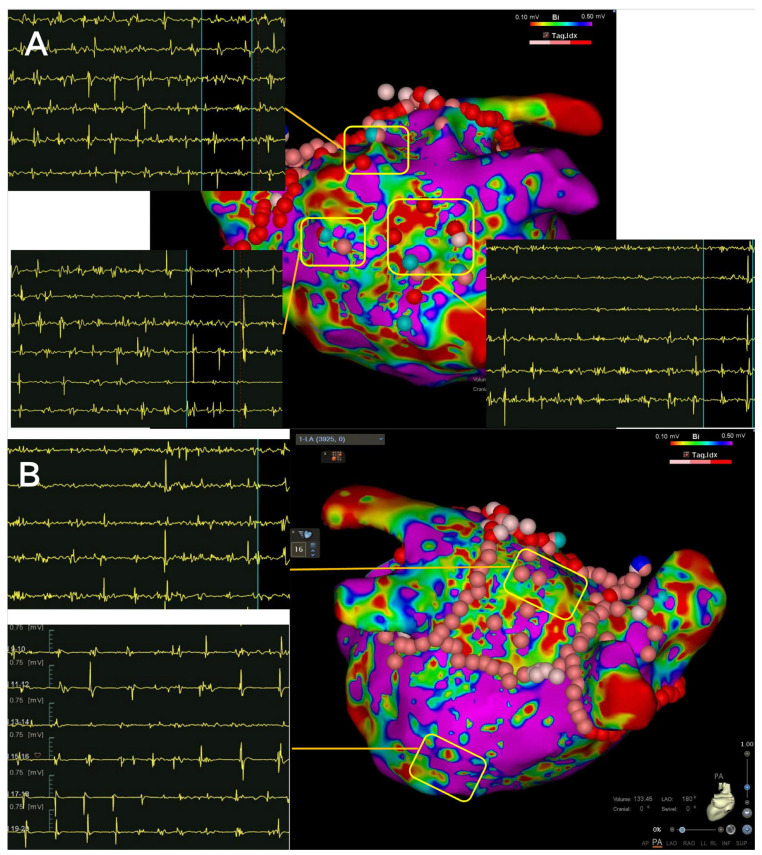
Modified Posterior Left Atrial Wall Isolation (MPWI) strategy used in this study. The ablation approach included pulmonary vein isolation (PVI), PWI and selective complex fractionated atrial electrograms (CFAEs) ablation in the roof and anterior wall of the left atrium. CFAEs in the anterior, roof, and posterior walls are shown in (**A**,**B**), while a relatively simple and discrete electrogram was recorded in the bottom section in (**B**).

All ablation procedures in this study were carried out as scheduled surgeries, with no emergency ablations performed. Prior to each procedure, a transesophageal echocardiography (TEE) was conducted on all patients. Endocardial 3D mapping was performed using multipolar mapping catheters with the CARTO (Biosense Webster, Irvine, CA, USA) system. Irrigated contact force-sensing ablation catheters were employed, maintaining a minimum target contact force of over 5 g. The recommended ablation targets for the CARTO system were an Ablation Index of 450 to 500 for the anterior region, and 350 to 400 for the posterior region. The PVI treatment involved wide antral circumferential ablation around the pulmonary veins, with electrical isolation as the endpoint. In patients undergoing MPWI procedures, after completing the PVI, posterior wall isolation (PWI) and selective complex fractionated atrial electrograms (CFAEs) ablation were performed on the roof and anterior wall. If PWI could not be achieved through linear ablation, mapping and targeting of the earliest electrograms within the “box” (the area on the posterior wall encircled by the posterior PVI lines on both sides, the roof ablation line, and the floor ablation line) were performed. Confirmation of PVI and PWI was achieved by identifying the entrance and exit blocks, and either spontaneous potentials or the complete absence of local electrograms, with no capture during high-output pacing. After achieving PVI and PWI, selective CFAEs in the roof and anterior wall of the left atrium were targeted (Figure 1). The CFAEs were characterized by fractionated potentials with multiple deflections from the isoelectric line (≥3 deflections) and/or potentials with continuous electrical activity without isoelectric lines, as previously described [18]. Additional linear ablation was only considered if corresponding atrial flutter was observed during the procedure. Ibutilide (1 mg) or amiodarone (70 mg) was administered intravenously to assist with cardioversion after PVI in the PVI group, and after PWI in the MPWI group. If atrial fibrillation persisted post-ablation, external cardioversion was performed.

#### Electrogram Analysis

**Figure 2:**
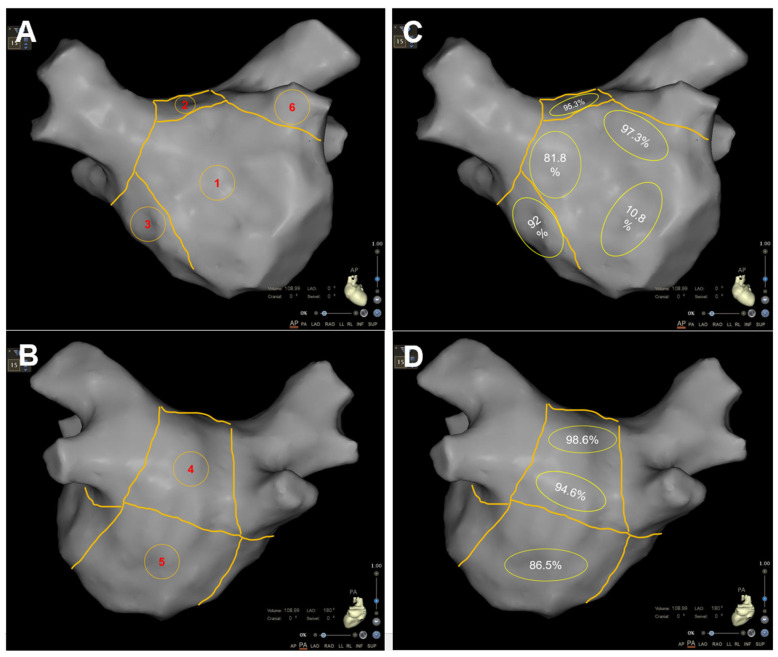
Anatomical division of the left atrium and CFAEs distribution. Panels (**A**,**B**) show anterior and posterior views of the left atrium, illustrating the 6-segment model. Panels (**C**,**D**) display the distribution of CFAEs in each LA segment, with the incidence of CFAEs in each section calculated and illustrated in (**C**,**D**): 1. Anterior Wall, 2. Roof, 3. Septum, 4. Posterior Wall, 5. Bottom, 6. Left atrial appendage (LAA).

Cycle lengths (CLs) in four PVs and various sections of the left atrium (LA) were recorded for all patients in atrial fibrillation (AF) during the ablation procedure. The LA was divided into six segments: anterior, posterior, roof, inferior, bottom, and septum (Figure 2). A multipolar catheter was sequentially placed in each PV (beyond the PV antrum) and in different segments of the LA for 10 s. The average CL was determined by manually measuring 30 consecutive CLs using calipers and expressed in milliseconds. The dominant wavefront was identified by reviewing all electrograms, and a representative, reliable electrogram of the wavefront was chosen, allowing consistent measurements across all 30 beats, which were annotated to determine the CL. High-frequency activation (HFA) patterns were classified based on the region with the highest frequency activation as follows: Type A: HFA observed among four PVs; Type B: HFA detected in the posterior, roof, or anterior wall; Type C: HFA detected in the bottom or septal wall; and Type D: similar HFA seen across most LA segments.

### 2.3. Low Voltage Surface Area and CFAEs Analysis

Voltage mapping was conducted while patients were in atrial fibrillation. Bipolar voltage was defined as the peak-to-peak voltage of the electrogram. The LA was segmented into anterior, posterior, roof, inferior, bottom, and septum sections for analysis. A low voltage area (LVA) was characterized as a contiguous region comprising at least 3 mapping points with a bipolar voltage < 0.5 mV. The dimensions of these low voltage regions within each predefined segment were manually measured using the surface area measurement tool on the CARTO system. The LVA index was calculated using the formula: total LA LVA/LA volume, with the volume determined via the CARTO system’s measurement tool. Additionally, the distribution of CFAEs in each left atrial segment was explored in this study.

### 2.4. Post Procedural Management and Follow-Up

All patients were monitored with ECGs following the procedure until hospital discharge and were subsequently followed up through cardiology outpatient visits. Oral anticoagulation was prescribed for at least 3 months post-procedure, with further treatment based on the CHA_2_DS_2_-VASc score. Antiarrhythmic drugs were either resumed or newly prescribed at the physician’s discretion. Clinic ECGs and 24-h Holter monitoring were conducted at 1 month, 3 months, 6 months, and 12 months after the procedure. Additional visits were scheduled if patients experienced symptoms. All antiarrhythmic medications were discontinued at the 3-month visit. Recurrent atrial arrhythmia was defined as any episode of sustained AF or AT lasting ≥ 30 s, excluding the initial 3-month blanking period, over a minimum of 12 months’ follow-up.

### 2.5. Statistical Analysis

Continuous variables were presented as means and standard deviations, except in cases where the distribution was skewed, in which medians and interquartile ranges (IQRs) were reported. Categorical variables were expressed as frequencies and percentages. To compare variables between 2 groups, the χ^2^ test or Fisher’s exact test was used for categorical data, while the t-test or Mann-Whitney U test was applied for normally distributed and skewed continuous data, respectively. For comparing non-normally distributed continuous variables across 3 or more groups, the Kruskal-Wallis test was utilized. A 2-sided *p* value of less than 0.05 was considered statistically significant.

Kaplan-Meier estimates were used to construct arrhythmia-free survival curves, with group comparisons performed using the log-rank test.

Cox proportional hazards regression was used to identify predictors of atrial arrhythmia recurrence. Variable selection for the multivariable model was based on the Bayesian Information Criterion (BIC). Hazard ratios with 95% confidence intervals were provided for the selected predictors.

All statistical analyses were conducted using R software (version 4.3.1).

## 3. Results

### 3.1. Study Patients

**Figure 3:**
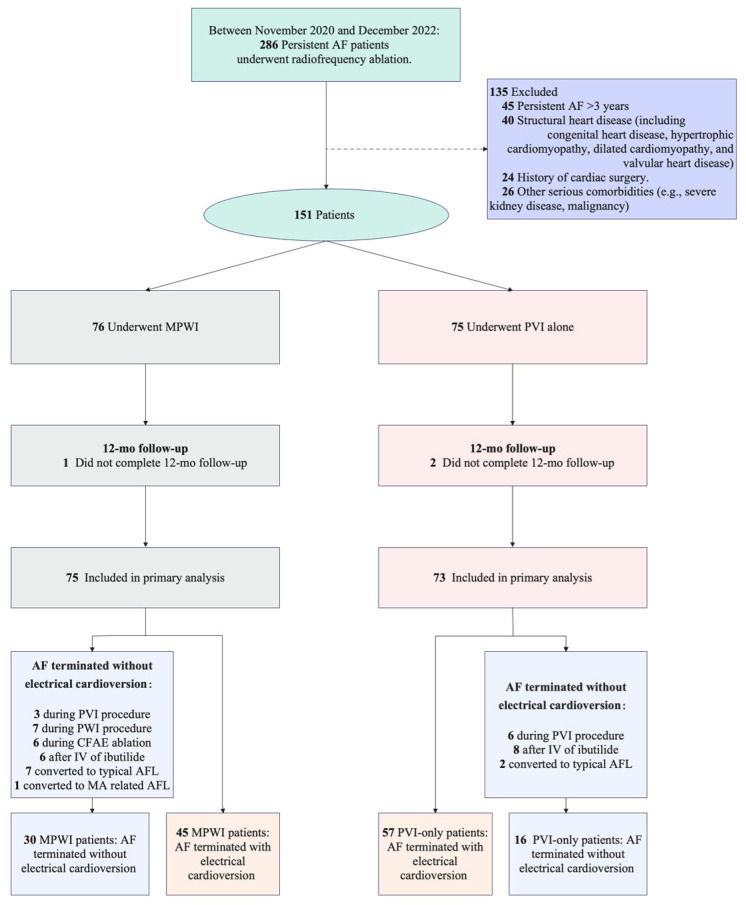
Consort diagram of study participants. PVI: pulmonary vein isolation; MPWI: modified posterior wall isolation; CFAEs: complex fractionated atrial electrograms; MA: mitral annulus.

From November 2020 to December 2022, a total of 151 patients were enrolled in the study. Of these, 148 (98.0%) successfully completed the 12-month follow-up. One patient in the MPWI group and two in the PVI-only group were lost to follow-up after 6 months, leading to their exclusion from the final analysis. As a result, efficacy outcomes were evaluated for 75 patients in the MPWI group, and 73 in the PVI-only group (Figure 3). Baseline characteristics for both groups are detailed in Table 1.

### 3.2. Procedural Characteristics

All patients achieved successful PVI. In the MPWI group, PWI was successfully completed in all patients, with 64% (48/75) requiring focal ablation within the posterior wall box to attain electrical isolation or silence. The distribution of CFAEs across different sections of the LA is shown in Figure 2. Any AF was terminated without the need for electrical cardioversion during the ablation procedure in 30 patients in the MPWI group (3 during the PVI procedure, 7 during the PWI procedure, 6 during CFAE ablation, 6 after intravenous ibutilide, 7 converted to typical AFL, 1 converted to MA-related AFL) and in 16 patients in the PVI group (6 during the PVI procedure, 8 after intravenous ibutilide, 2 converted to typical AFL).

### 3.3. Primary Efficacy Outcome

**Figure 4:**
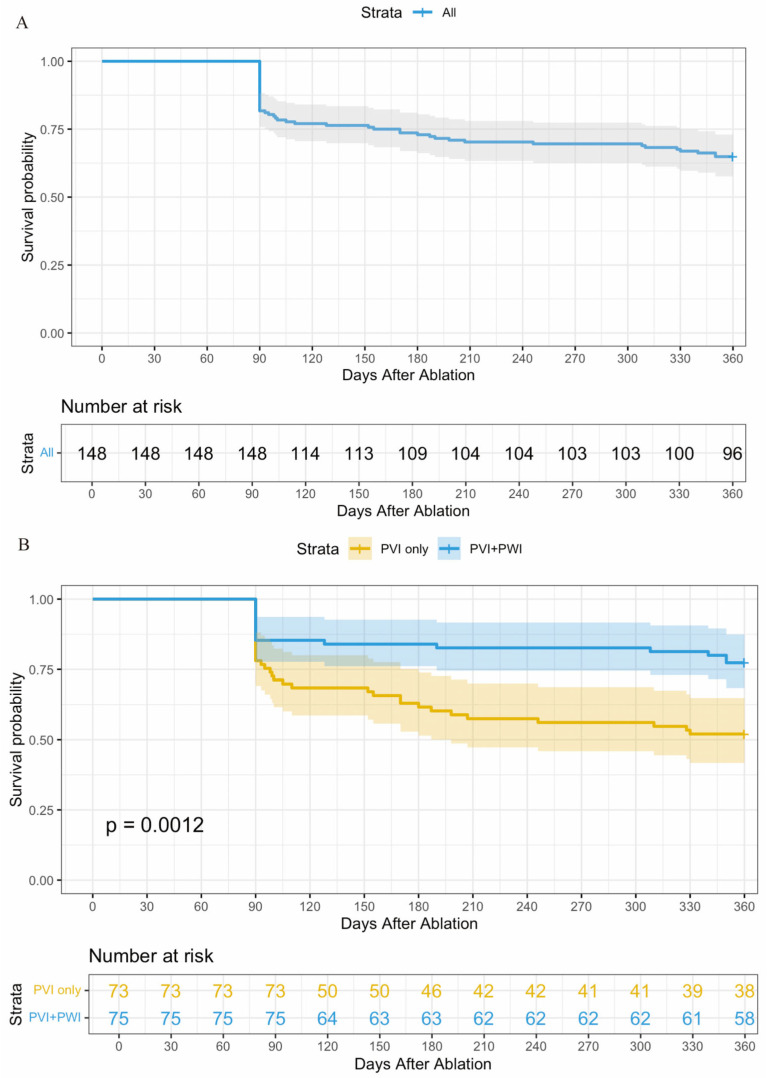
Kaplan-Meier Estimate of Arrhythmia-free Survival in PeAF patients with different ablation strategies. (**A**): Kaplan-Meier Estimate of Arrhythmia-free Survival for the entire cohort; (**B**): Kaplan-Meier Estimate of Atrial Arrhythmia-free Survival for each group. PVI: pulmonary vein isolation; MPWI: modified posterior wall isolation.

At the 12-month follow-up, 96 out of 148 patients (64.9%) across the entire cohort remained free from atrial arrhythmia after a single ablation procedure, without the need for antiarrhythmic medication. Specifically, 77.3% (58 out of 75) of patients in the MPWI group achieved this outcome, compared to 52.1% (38 out of 73) in the PVI-only group (*p* = 0.001) (Figure 4). No significant adverse events, such as mortality, cardiac tamponade, or atrioesophageal fistula, were reported in either study group.

### 3.4. High Frequency Pattern for LA

**Figure 5:**
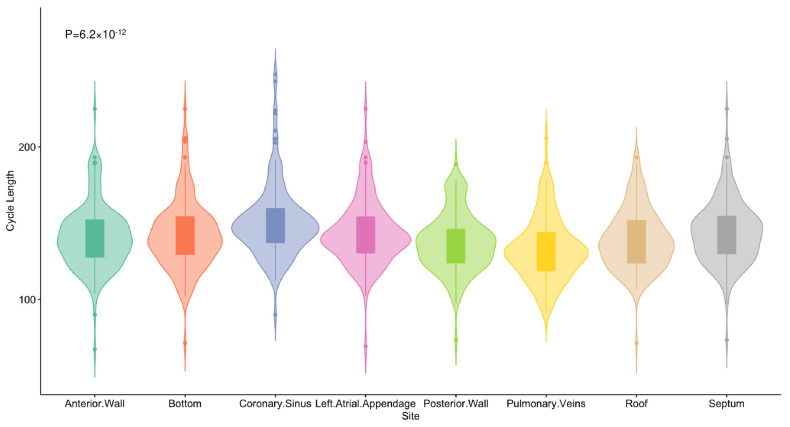
High frequency pattern of the cohort. The activation frequency for the CS and each LA segment was compared, showing that ACF recorded in the CS was significantly lower than in the LA segments, with no significant difference among the LA sections.

The activation frequency (ACF) recorded in the CS was notably lower than that in various sections of the LA. While no significant differences in ACF were found among the different LA segments, the distribution of HFA patterns varied significantly between types (*p* < 0.001) (Figure 5). Type A and Type B together accounted for the majority of cases, with 45.27% for Type A and 41.89% for Type B. In contrast, Type C represented only 9.46%, and Type D was the least common, with just 3.38% (Table 2).

### 3.5. Recurrence Predictors Based on the BIC Model

A Cox regression analysis with backward elimination, guided by the Bayesian Information Criterion (BIC), was conducted to identify key predictors of atrial arrhythmia recurrence. The initial model for the entire cohort included the ablation strategy and various clinical and echocardiographic parameters: gender, age, AF duration, BMI, hypertension, diabetes, hyperlipidemia, coronary heart disease, heart failure, stroke, smoking, alcohol consumption, aortic root diameter, LAD, LVIDd, LVIDs, PAP, EF, RAD, mitral regurgitation level, tricuspid regurgitation level, LAV, high-frequency activation pattern, and LVA index. For subgroup analyses of the MPWI and PVI-only groups, the models included the same variables except for ablation strategy, as it was uniform within each group.

Using the BIC-based stepwise Cox regression analysis, two significant predictors of atrial arrhythmia recurrence were identified for the entire cohort: ablation strategy and AF duration. The MPWI group significantly lowered the risk of recurrence compared to the PVI-only group (HR = 0.418, 95% CI: 0.234–0.747, *p* = 0.003), while a longer AF duration increased the risk of recurrence (HR = 1.009, 95% CI: 1.005–1.012, *p* < 0.001). (Table 3).

In the MPWI group, the BIC-based Cox regression identified three independent predictors of recurrence: AF duration (HR = 1.013, 95% CI: 1.008–1.018, *p* < 0.001), LVIDs (HR = 1.173, 95% CI: 1.023–1.346, *p* = 0.023), and moderate or greater tricuspid regurgitation (HR = 5.651, 95% CI: 1.841–17.348, *p* = 0.003). All three factors significantly increased recurrence risk, with tricuspid regurgitation showing the strongest association, followed by LVIDs and AF duration (Table 3).

In the PVI group, the BIC-based Cox regression identified the LA LVA index (HR = 1.005, 95% CI: 1.001–1.008, *p* = 0.005) as the sole significant predictor of atrial arrhythmia recurrence, indicating that an increase in this parameter was strongly associated with a higher risk of recurrence (Table 3).

## 4. Discussion

### 4.1. Major Findings

The key results of this study were: (1) The combination of PWI and selective CFAEs ablation with PVI significantly improved arrhythmia-free survival at 12 months compared to PVI alone. (2) In the MPWI group, AF duration, LVIDs, and moderate or greater tricuspid regurgitation were independent predictors of atrial arrhythmia recurrence. (3) In the PVI group, the LVA index was an independent predictor of atrial arrhythmia recurrence.

### 4.2. LA LVA and Ablation Results

Atrial fibrosis is a hallmark of structural remodeling and contributes to the initiation and persistence of AF through mechanisms such as conduction anisotropy, conduction heterogeneity, and re-entry [19,20]. Low-voltage areas (LVAs) in the atrium are often used as indicators of atrial fibrosis. These areas, characterized by reduced electrical activity and typically defined by a bipolar voltage of less than 0.5 mV, correspond to regions of fibrotic tissue with reduced electrical conduction. The extent of LVAs, often measured by the LVA index, provides an indirect assessment of atrial fibrosis and structural remodeling. Several studies have explored the role of LVAs in guiding ablation strategies for persistent AF. For example, the ERASE-AF trial [21] (Low-Voltage Myocardium-Guided Ablation Trial of Persistent Atrial Fibrillation) used 3D mapping to identify LVAs and reported improved arrhythmia outcomes in patients who underwent LVA-targeted ablation compared to those who received PVI alone. However, other large randomized studies, including the CAPLA study, showed no reduction in arrhythmia recurrence when LVAs were targeted compared to PVI alone. The negative results of the CAPLA study were attributed to the more extensive atrial substrate, beyond just the posterior wall. Additionally, in most studies, LVAs were identified using voltage mapping during sinus rhythm (NSR), which may underestimate the atrial substrate for AF, as some tissue may experience electrical remodeling without structural changes. Tissue with voltage between 0.5–1.5 mV may still serve as a substrate for sustaining AF. A previous study [22] revealed no anatomical correlation between CFAE sites and LVAs during NSR. Norman [23] reported that the correlation between low-voltage areas and posterior LA MRI-DE is significantly stronger when acquired during AF compared to sinus rhythm (SR). In this study, the atrial substrate was reassessed during AF rather than SR. Subsequent risk factor analysis confirmed that the LVA index is an independent predictor of atrial arrhythmia recurrence in the PVI group, consistent with previous studies. In the MPWI group, the BIC model identified AF duration, LVIDs, and moderate or greater tricuspid regurgitation as independent predictors of recurrence. This suggests that while the ablation strategy may alter the LA substrate to some extent, other factors beyond the LA substrate also play significant roles in recurrence. The strong link between tricuspid regurgitation and recurrence in this group is particularly notable, suggesting a potential role for right atrium remodeling in AF recurrence following extensive LA ablation. Masamichi [24] similarly found that severe tricuspid regurgitation was associated with AF recurrence after PVI in patients with a normal LA. Tricuspid regurgitation was believed to promote right atrial substrate abnormalities, leading to right-sided non-PV triggers that were not addressed during the procedure, which may explain the higher recurrence rates in patients with tricuspid regurgitation in the MPWI group.

The identification of different recurrence predictors for each ablation strategy offers insight into the mechanisms of AF recurrence after ablation. In the PVI-only group, the LA LVA index being the sole predictor suggests that residual LA substrate is a key factor in recurrence when only PVI is performed. On the other hand, the MPWI group displayed a more complex set of predictors, including AF duration, LVIDs, and tricuspid regurgitation. This indicates that while the MPWI approach may address some LA substrate issues, factors such as AF chronicity, left ventricular function, and right heart conditions also play critical roles in determining outcomes. The strong association between tricuspid regurgitation and recurrence in the MPWI group is particularly intriguing and highlights the need for further investigation into the role of right atrium remodeling in AF recurrence after extensive LA ablation.

### 4.3. High Frequency Activation Pattern and CFAEs of LA

This study also examined the high-frequency activation (HFA) patterns of the LA. A substudy of CAPLA [25] demonstrated that rapid PW activity is linked to a higher risk of AF recurrence following catheter ablation. The inclusion of PWI in that subgroup led to a significant improvement in maintaining AF-free status compared to PVI alone. In this study, the activation frequency across different LA sections was further analyzed, revealing that the majority of HFA was concentrated in the PVs, posterior wall, roof, and anterior wall. This suggests that different LA regions play distinct roles in sustaining PeAF, which explains why no additional ablation was performed on the bottom and septum in the MPWI group. Although the HFA patterns were not significantly associated with arrhythmia recurrence in the Cox regression analysis, this may be due to the complex electrophysiological mechanisms driving PeAF, which involve not only high-frequency rotors, but also functional and structural reentry circuits that remain poorly understood. A further analysis of atrial potentials showed a widespread distribution of CFAEs across all LA sections, in line with previous studies [26]. To better address the LA substrate beyond PVI+PWI while avoiding excessive LA ablation, CFAEs in the roof and anterior wall were specifically targeted, rather than modifying the entire LA LVA/CFAE regions, as the majority of HFA was located in these sections. This ablation strategy led to a significant reduction in atrial arrhythmia occurrence compared to PVI alone. The CFAE regions are often considered key for sustaining AF, as they are thought to correspond to critical pivot points or rotors [27,28]. Ablating CFAE sites can result in the slowing, regularization, and even termination of AF, with these acute changes potentially correlating with long-term sinus rhythm maintenance [29,30,31]. In this study, AF termination during the ablation procedure occurred in [30] cases in the MPWI group, which was significantly higher than in the PVI group.

## 5. Conclusions

In patients undergoing first-time catheter ablation for persistent AF, the addition of PWI and selective CFAE ablation to PVI significantly improved atrial arrhythmia-free outcomes at 12 months compared to PVI alone. In the PVI group, the LA LVA index was the only independent risk factor for atrial arrhythmia recurrence. In the MPWI group, AF duration, LVIDs, and moderate or greater tricuspid regurgitation were identified as independent predictors of recurrence. These results highlight the different mechanisms underlying arrhythmia recurrence in the two ablation strategies, highlighting the need for personalized approaches in managing persistent AF. Our study supports the inclusion of PWI and selective CFAE ablation with PVI to improve long-term outcomes.

## 6. Limitation

First, as a single-center retrospective study with a relatively small sample size, our findings may have limited generalizability and statistical power. Larger prospective randomized studies are necessary to confirm these conclusions. Second, the atrial substrate in sinus rhythm was not evaluated due to the specific ablation strategy used, and CFAEs were assessed subjectively by the operator, which may introduce measurement bias. Manual measurement and calculation of the shortest CL also present challenges in integrating them into procedural workflow. Developing an automated algorithm could address the practical difficulties of manual real-time assessment and reduce interobserver variability. Third, the primary endpoint was primarily assessed via ECG and 24-h Holter monitoring, rather than an implantable loop recorder, which may have led to an underestimation of atrial arrhythmia recurrence in this study. Fourth, we did not analyze data on procedure times or fluoroscopic times between the groups. As longer procedure and fluoroscopic times are potential drawbacks of the ablation strategy, this represents a limitation in our ability to fully evaluate and compare the two strategies in a neutral manner. Future studies should incorporate these measurements to provide a more comprehensive comparison of treatment strategies.

## Figures and Tables

**Table 1:** Baseline characteristics.

	MPWI	PVI Alone	
Characteristic	n = 75	n = 73	*p* Value
Demographics			
Age, median (IQR), y	64 (55, 69)	63 (56, 68)	0.77
Men	56 (74.7)	53 (72.6)	0.78
BMI, median (IQR)	25.35 (23.92, 26.7)	25.65 (24.22, 27.68)	0.41
Time from first onset of AF, median (IQR), month	18 (6, 54)	24 (12, 60)	0.13
Long-term AF (≥6 Months)	60 (80.0)	64 (87.7)	0.21
Smoking	22 (29.3)	18 (24.7)	0.52
Drinking	14 (18.7)	8 (11)	0.19
Comorbidities			
Hypertension	45 (60)	43 (58.9)	0.89
Type 2 diabetes	9 (12)	15 (20.5)	0.16
Hyperlipidemia	12 (16)	6 (8.2)	0.15
Coronary heart disease	12 (16)	7 (9.6)	0.24
Heart failure	11 (14.7)	7 (9.6)	0.35
Stroke	7 (9.3)	12 (16.4)	0.20
Echocardiographic Parameters			
Aortic root diameter, median (IQR), mm	35 (32, 37.5)	34 (31, 37)	0.15
LAD, median (IQR), mm	46 (44, 48.5)	45 (44, 48)	0.81
LVIDd, median (IQR), mm	49 (47, 51)	49 (46, 52)	0.80
LVIDs, median (IQR), mm	31 (30, 33)	31 (30, 35)	0.79
Ventricular septal thickness, median (IQR), mm	9 (9, 10)	10 (9, 10)	0.26
PAP, median (IQR), mmHg	33 (30.5, 35)	33 (30, 37)	0.66
LVEF, median (IQR), %	64 (61, 67)	64 (60, 66)	0.22
RAD, median (IQR), mm	59 (55, 61)	57 (54, 62)	0.45
LAV, mL	152.41 (30.75)	156.86 (33.23)	0.40
LVA index *	15.60 (7.83, 22.86)	13.10 (6.76, 20.00)	0.11
Moderate or greater mitral regurgitation	10 (13.33)	13 (17.81)	0.60
Moderate or greater tricuspid regurgitation	16 (21.33)	19 (26.03)	0.63
Laboratory Parameters			
Hemoglobin, median (IQR), g/L	146 (135.5, 156)	146 (135, 157)	0.84
Potassium (K), median (IQR), mmol/L	3.9 (3.7, 4.2)	4 (3.8, 4.2)	0.21
Sodium (Na), median (IQR), mmol/L	142 (141, 144)	142 (141, 143)	0.44
Chloride (Cl), median (IQR), mmol/L	105 (104, 107)	105 (104, 106)	0.14
Cardiac Troponin T (cTnT), median (IQR), ng/mL	0.009 (0.006, 0.01)	0.009 (0.007, 0.012)	0.15
NT-proBNP, median (IQR), pg/mL	733 (464.65, 1117)	584 (389, 940)	0.03

Values are presented as mean ± SD for variables following a normal distribution, median (Q1, Q3) for variables not following a normal distribution, and n (%) for categorical variables. Abbreviations: AF = Atrial Fibrillation; BMI = Body Mass Index; IQR = Interquartile Range; LAD = Left Atrial Diameter; LAV = Left Atrial Volume; LVEF = Left Ventricular Ejection Fraction; LVIDd = Left Ventricular Internal Diameter in Diastole; LVIDs = Left Ventricular Internal Diameter in Systole; PAP = Pulmonary Artery Pressure; PVI = Pulmonary Vein Isolation; PWI = Pulmonary Vein Isolation with Posterior Wall Isolation; RAD = Right Atrial Diameter. * The LVA index was calculated using the formula: total extent of LA LVA/LA volume.

**Table 2:** Distribution of High Frequency by Electrophysiological Cycle Length.

Ablation Site Classification	Number of Cases	Percentage (%)	Χ^2^ Value	*p*-Value
Pulmonary Vein Potentials	67	45.27	83.189	<0.001
Left Atrial Septum and Bottom Potentials	14	9.46		
Anterior Wall, Roof, and Posterior Wall Potentials	62	41.89		
Other (Uniform Potentials, etc.)	5	3.38		
Total	148	100		

Table 2 High-frequency activation patterns were categorized based on the region of highest frequency activation (HFA). Type A: HFA observed among 4 PVs; Type B: HFA observed in the posterior, roof, or anterior wall; Type C: HFA observed in the bottom or septal wall; and Type D: Similar HFA observed across most LA segments. The proportions of these different HFA patterns were compared, with Type A and Type B being the most prevalent. Type C accounted for only 9.46%, while Type D was the least represented at 3.38%.

**Table 3:** Recurrence predictors based on the BIC Model.

Group	Factor	HR	95% CI	*p* Value
Entire Cohort	Ablation Strategy (MPWI)	0.418	0.234–0.747	0.003
Entire Cohort	AF Duration	1.009	1.005–1.012	<0.001
MPWI	AF Duration	1.013	1.008–1.018	<0.001
MPWI	LVIDs	1.173	1.023–1.346	0.023
MPWI	Moderate or greater tricuspid regurgitation	5.651	1.841–17.348	0.003
PVI only	LA LVA Index	1.005	1.001–1.008	0.005

## Data Availability

Data are contained within the article.

## Supplementary Materials

The following supporting information can be downloaded at: https://www.mdpi.com/article/10.3390/jcdd11100308/s1, Table S1: Detailed Inclusion and Exclusion Criteria; Text S1: Echocardiographic Parameters and Mitral/Tricuspid Regurgitation Assessment.
