# Supplementary material for: Effect of Pulmonary Vein Isolation with Left Atrial Wall Isolation Plus Selective CFAE Ablation in Patients with Persistent Atrial Fibrillation
Source: J Cardiovasc Dev Dis. 2024 Oct 4;11(10):308. doi: 10.3390/jcdd11100308 (PMC11508299; doi:10.3390/jcdd11100308)
Supplement: Supplementary file 1 [file jcdd-11-00308-s001.zip › jcdd-3154439-supplementary.pdf]

Table S1. Detailed Inclusion and Exclusion Criteria:

Inclusion Criteria:

- Patients aged 18 years or older;
- First-time catheter ablation procedure for atrial fibrillation;
- Patients with persistent AF, defined as a sustained episode lasting more than 7 days and less than 3 years;
- Symptomatic AF that is refractory to at least one antiarrhythmic medication, with symptoms such as palpitations, shortness of breath, chest pain, fatigue, left ventricular dysfunction, etc.;
- At least one documented episode of persistent AF within the last 2 years by ECG or Holter;
- Ability and willingness to provide written informed consent and comply with all peri-ablation and follow-up requirements.

Exclusion Criteria:

- Patients with paroxysmal AF (sustained episode lasting less than 7 days);
- Patients with long-standing persistent AF (lasting more than 3 years);
- Patients with a history of cardiac surgery;
- Structural heart disease (Ischemic cardiomyopathy, hypertrophic cardiomyopathy, dilated cardiomyopathy, congenital heart disease and valvular heart disease.);
- History of atrial septal repair or atrial myxoma;
- Severe liver or kidney disease, malignancy, or terminal illness;
- Other conditions deemed inappropriate for the study by the investigators.

Text S1: Echocardiographic Parameters and Mitral/Tricuspid Regurgitation Assessment.

Echocardiographic Parameters

All echocardiographic parameters were measured using transthoracic echocardiography with a standard echocardiographic machine. The aortic root diameter was measured in the parasternal long-axis view at end-diastole. The left atrial diameter (LAD) was assessed in the apical four-chamber view at end-diastole. Left ventricular internal diameter in diastole (LVIDd) and systole (LVIDs) were measured in the parasternal long-axis view. Ventricular septal thickness was evaluated at the level of the interventricular septum in the parasternal short-axis view.

Pulmonary artery systolic pressure (PASP) was estimated using Doppler echocardiography by measuring the peak velocity of tricuspid regurgitation and applying the Bernoulli equation. Additionally, inferior vena cava size and respiratory variation were assessed to estimate right atrial pressure.

The left ventricular ejection fraction (LVEF) was calculated using the biplane method of discs (Simpson's rule) or other 2D echocardiographic methods. Right atrial diameter (RAD)

was measured in the apical four-chamber view at end-diastole. Left atrial volume (LAV) was assessed using the biplane method of discs (Simpson's rule) in apical four-chamber and two-chamber views at ventricular end-systole, or using 3D echocardiography.

Right ventricular function was evaluated by measuring tricuspid annular plane systolic excursion (TAPSE) and right ventricular systolic peak velocity (S'). When necessary, right ventricular fractional area change (FAC) was calculated.

Left ventricular diastolic function was assessed by measuring mitral inflow velocities (E and A waves), tissue Doppler imaging (e' velocity), E/e' ratio, and left atrial volume index.

Mitral and Tricuspid Regurgitation Assessment
Mitral and tricuspid regurgitation were evaluated using Doppler echocardiography with color flow mapping, continuous-wave Doppler, and pulsed-wave Doppler techniques.

Mitral Regurgitation (MR)**
Measurement: Color Doppler echocardiography was used to assess the jet area of mitral regurgitation, with the severity classified based on the width and extent of the color flow jet. The jet's origin and its impact on the left atrium were also evaluated. Vena contracta width was measured, and when necessary, effective regurgitant orifice area (EROA) and regurgitant volume were calculated.
Classification: MR severity was classified into mild, moderate, and severe based on the jet's width, the area of the regurgitant flow, vena contracta width, EROA, regurgitant volume, and its effect on the left atrial volume and left ventricular function.

Tricuspid Regurgitation (TR)
Measurement: Color Doppler echocardiography was used to visualize the tricuspid regurgitant jet. The peak velocity of the regurgitant jet was measured using continuous-wave Doppler echocardiography, and the severity was assessed based on the pressure gradient and the width of the jet. Vena contracta width was also measured.
Classification: TR severity was classified as mild, moderate, or severe. This classification is based on the regurgitant jet's area, the maximal pressure gradient across the tricuspid valve, vena contracta width, and the impact on right atrial and ventricular dimensions. For severe TR, inferior vena cava size and respiratory variation were also evaluated.
